# A Simulation Model for Intra-Urban Movements

**DOI:** 10.1371/journal.pone.0132576

**Published:** 2015-07-10

**Authors:** Nimrod Serok, Efrat Blumenfeld-Lieberthal

**Affiliations:** The David Azrieli School of Architecture, Yolanda and David Katz Faculty of the Arts, Tel Aviv University, Ramat Aviv, Tel-Aviv, 69978, Israel; University of Gävle, SWEDEN

## Abstract

Human mobility patterns (HMP) have become of interest to a variety of disciplines. The increasing availability of empirical data enables researchers to analyze patterns of people’s movements. Recent work suggested that HMP follow a Levy-flight distribution and present regularity. Here, we present an innovative agent-based model that simulates HMP for various purposes. It is based on the combination of regular movements with spatial considerations, represented by an expanded gravitation model. The agents in this model have different attributes that affect their choice of destination and the duration they stay in each location. Thus, their movement mimics real-life situations. This is a stochastic, bottom-up model, yet it yields HMP that qualitatively fit HMP empirical data in terms of individuals, as well as the entire population. Our results also correspond to real-life phenomena in terms of urban spatial dynamics, that is, the emergence of popular locations in the city due to bottom-up behavior of people. Our model is novel in being based on the assumption that HMP are space-dependent as well as follow high regularity. To our knowledge, we are the first to succeed in simulating HMP not only at the inter-city scale but also at the intra-urban one.

## Introduction

Recently, human mobility patterns (HMP) have become of interest to a variety of disciplines. This is due to the importance of understanding HMP as the basis for developing epidemiology models, mobile wireless network planning, urban and transportation planning, and more [1–7]. In addition to the growing interest in this area, the increasing availability of empirical data that tracks human trajectories enables researchers to analyze accurately emerging patterns in these movements.

The classic models of HMP suggested that it can be simulated by Brownian motion or random walk [3, 4, 8]. This means that the human movements were assumed to follow a successive number of random steps, in terms of distance and rotation. More recent work that was based on the analysis of empirical data, gained from tracking the location of numerous bank notes over time, questioned this classic perception and suggested that HMP follow a Levy-flight distribution [9]. This means that the distances distribution of the trajectories follows a power law. In other words – there are many short trajectories and very few considerably long ones. This work was followed by [10] who analyzed the trajectories of 100,000 mobile phone users over a six-month period. Their findings suggest that HMP follow Levy-flight and show a high degree of regularity, in time and space. These findings were also found by [11] who used GPS data and by [12] who analyzed the data of 50,000 mobile-phone users over a period of 3 months. This means that people have a routine of returning to several specific locations.

More recently, [13] analyzed data, obtained from 50 taxicabs who conducted 72,000 trajectories during a six-month period. Their findings suggest that the HMP indeed follow the Levy-flight behavior. They explained this as largely connected to the topology of the underlying street network. [14] Analyzed the trajectories of 258 volunteers who attached a GPS device to their car for about a week. They found that the HMP are regular and have scaling properties (Levy-flight). According to their work, this behavior was governed by the scaling and hierarchical properties of the destination clusters and by the individual preferences of the people in the examined sample.

Another work [15] analyzed the movements of 925,030 Foursquare [16] users over a period of about 6 months. Their results also support the assumption that HMP follow a power law distribution (Levy-flight). However, unlike previous work, they suggested that this is due to the different motives that drive one’s decision to move. They also discovered that the probability of moving from one place to another is dependent on the number of intervening opportunities rather than the physical distance between them. Despite the obvious importance of this field, it appears that there are only a few theoretical models that mimic HMP; in their work, [17] proposed a dynamical task-based model to investigate the origin of non-Poisson properties in human mobility patterns. Another model [18] was developed based on heterogeneous centrality and overlapping community structure in social networks. Recently, [19] presented a three-scale mobility cost-benefit model for human displacements based on simple geometrical constrains, and [15] developed a rank-based movement model for human movements in cities.

More recently, [20] suggested that HMP are liable to a universal law, i.e. independent of spatial characteristics. They presented a universal model for HMP, called the radiation model, and showed that it corresponds with empirical data. However, the universality of this model was questioned by [21] who reinforced the importance of spatial parameters in HMP. By doing so, they re-validated the role of gravitation (the importance of size and distance) on HMP. Both of the above models failed to describe HMP within the city’s scale.

The presented model is a spatially embedded agent based model (ABM). An ABM is a method to compute simulations of complex systems, where each agent represents an autonomous entity that acts based on predetermined rules [22–25]. Following these rules, the agents evaluate situations in their modelled world and make decisions. In spatially embedded models, the infrastructure of the model represents space and the decisions of the agents take under consideration spatial issues such as distance, land uses etc. [26, 27]. Cities are complex system by their nature [28–30]. They have developed as a result of the decisions and actions of many actors who were motivated by numerous ambitions as well as political, cultural, socio-economic and other considerations. Thus, in the last decades, ABMs in general and spatially embedded ABMs in particular have become a common methodology to explore urban issues [26, 28, 29, 31–35].

Our model is based on the combination of regular movements that can be found in people’s daily/weekly/monthly routine, combined with spatial considerations that are represented by an expanded gravitation model (see Eq 1). It is a stochastic, bottom-up model that follows the different behaviors of individual agents over a period of one month. The movement of the agents in our model mimics real-life situations by following some basic parameters such as age, employment status, marital status, location, and the time of the day. The agents in the model have different attributes, preferences and commitments that affect their choice of destination and the duration they stay in each location. Despite the stochastic characteristics of the model, and it being a bottom-up model, the HMP it yields correspond to empirical data, found in [10, 13, 14]. In its current configuration it is calibrated with the center of the city of Tel Aviv in terms of size and land uses, and samples only 10,000 agents. However, by using different parameters, it can be adjusted to different urban areas and different social behavior as well. The results of the model suggest that we have succeeded in creating a complex, self-organizing system that mimics human mobility patterns in real urban environments. The presented study based on a computer simulation model (written in C# computer language and run on the.Net framework), does not aim to predict the movement of people or transportation systems. It cannot be applied to real cities or forecast how each urban agent will move. This is, however, not its purpose. As a simulation model, its essence is to identify and analyze the relationships between the variables, their correlations, catalysts and mutual influences and so on. The results of this model are not valid in regards to real cities, yet, the insights that can be obtained from it are.

The remainder of the paper is organized as follows: in section 2 we explain the spatial setting we use to simulate urban environment. In section 3 we present the agent-based model for simulating human mobility patterns, and in section 4 we present the results of the above model and compare them to real-world empirical data. We close with a discussion and some concluding remarks.

## Spatio-Temporal Definitions of the Model

### Space

Our proposed model is a spatially-embedded one. Thus, it was important for us to specifically define the spatial environment we run our agents on. For that, we have decided to use an environment that resembles a real modern city on the one hand, yet simplify its complexity on the other. We developed a methodology which is used to construct the spatial basis of our model, and used the city of Tel Aviv as a basis for the statistical properties. This methodology, however, can naturally be applied to other cities as well.

Our environment is based on a cellular grid, where each cell represents 25*25 square meters. The total size of the grid is 100*100 squares, representing an environment of 2,500*2,500 square meters. This area resembles the city center of Tel Aviv. Next, we defined six land uses: residential, employment, open public spaces, public buildings, entertainment and retail, and a mixed land use of residential, employment, and retail. To determine the mixture of land uses in the city as well as the typical sizes of clusters they occupy, we studied the case of Tel Aviv. Based on municipal plans and a comparison of these plans to reality (which was done as some of the plans had gone through spot-zoning amendments over time), we defined the percentage of each land use and the range of the typical sizes of their clusters. Then, we used these percentages to set a list of possible clusters, defined by their land uses and sizes, and arranged them randomly in space based on the range of possible clusters each land use comprises. This means that at each iteration a land-use was picked at random and allocated with a random cell as well as a size (chosen from the list described above). At the following iterations the cluster of this land use was enlarged by adding to it adjacent cells, until it reached its designated size. Then, another land use was picked at random from the list and the entire process is repeated, until no more clusters (and vacant locations) were left.

**Fig 1:**
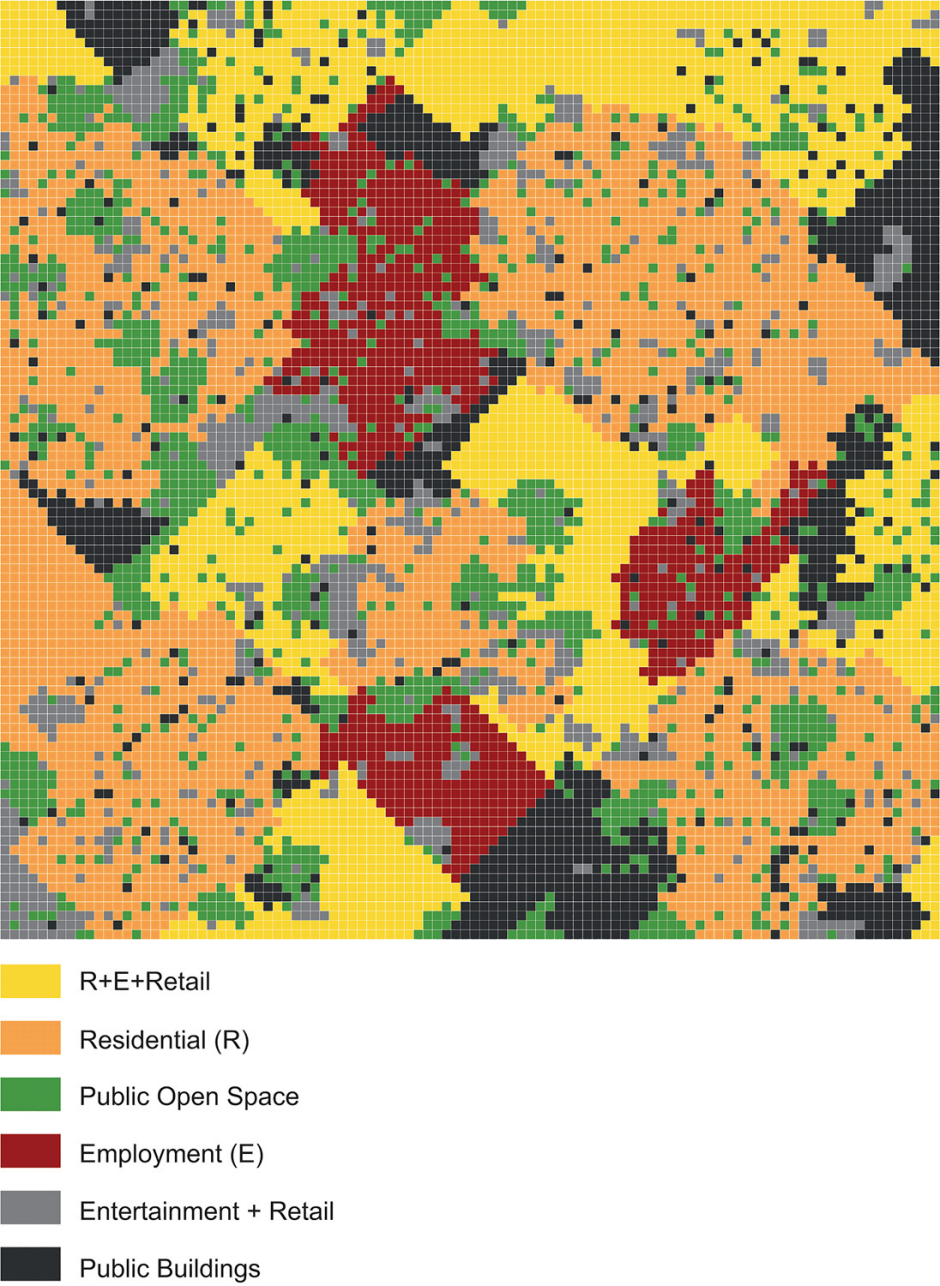
A typical example for land use spatial distribution in the simulated environment.

The distribution of clusters sizes was either normal or exponential, based on the analysis of the real data. Fig 1 presents a typical example of the simulated environment the above algorithm yields, while Table 1 presents the numerical parameters we used for our model. In order to simulate the real-life differences of attractiveness between different areas with the same land use, each cell *i* was randomly given a specific attractiveness value *θ*_*i*_. The specific attractiveness values of all the cells followed a normal distribution.

**Table 1:** Land uses distribution for the spatial environment used in our model.

Land Use	Cells	Percentage (%)	Size of Max Cluster
**Residential (R)**	3,400	34	1000
**Employment (E)**	1,000	10	500
**Public buildings**	1,200	12	200
**R + E + Retail**	2,400	24	300
**Entertainment and Retail**	800	8	40
**Public Open Space**	1,200	12	100

### Time

As people move differently at different times of the day and week, we divided the time in our model as follows: a week consists of 5 working days and a 2-day weekend, where a day is defined as 24 hours, divided into 6 periods: morning (6am-12pm), morning rush-hour (7:30am-9:30am), noon (12pm-5pm), evening rush-hour (5pm-7pm), evening (7pm-10pm), and night (10pm-6am). Each iteration in the model represents 15 minutes in reality. As we elaborate on in the next section, each time slot has a significant role in determining the agents’ nature of behavior.

## The Agent-Based Model for Simulating Human Mobility Patterns

There are 4 types of agents in our model that represent different types of people who share common characteristics, among them similar daily and weekly routines. These groups are based on the agents’ age and/or marital status: teenagers, bachelors, married people, and seniors. Each agent is characterized by the following attributes:

- Home address: each agent is assigned a home address which is randomly selected from all available locations of the *residential* or the *residential and employment* land uses.
- Employment status: students, married people, and seniors are either working or not (each groups is represented by a different percentage of working community).
- Work/school address: each of the teenagers and the students is given a school/university address which is randomly selected from all available locations of public buildings, and working agents are given a work location out of all available locations of the *employment* or the *residential and employment* land uses. For working agents, there is a low probability that their work location belongs to *public buildings* or *entertainment and retail* land uses.

Working/studying agents have a weekly routine of going to school/university/work at the morning of each weekday, but they leave these locations at different times (e.g. a teenager pupil leaves school earlier than a working agent leaves his work). During the afternoons and evenings each group follows a different behavior patterns – a family man usually returns home or stops at one location before heading there (for example – the grocery store) while a student might go from the university to work or to study with a friend or even to a pub after his day at the university ends. The senior group, on the other hand, is divided into working and retired people. The working seniors work less hours than the other working groups and the retired ones do not have a definite routine. This, of course, corresponds to real-life situations, where people follow routine behavior such as going to work/school, but occasionally they have other activities that interfere with their routine (e.g. running urgent errands). Additionally, as in real life, agents at retirement age either work less hours that younger ones, or do not work at all. In the next section we explain the algorithm that governs the agents’ movements, based on their characteristics and their spatio-temporal location.

### Agents’ movement

People mobility patterns can be roughly divided into regular and irregular movements. The regular movements depend on their routines while the irregular ones can be either to places they visit once (e.g. jury duty) or to places they return to on an irregular basis (for example, a restaurant one likes or the theater). To simulate these two behaviors the movement of the agents in the model depends on their type, their current location, and on the time of day and the day in the week.

Our algorithm is divided into two stages; in the first stage it is decided if the agent is going to move or stay put. If the agent is to move, his destination is set in the second stage. Appendices A and B in S1 File describe the algorithms of these stages. This model was designed to examine HMP in the scale of a city center only. For this reason, only movements that both their origins and destinations were limited to the model space (i.e. 2,500*2,500 square meters, as elaborated in section 2) were modeled. In this model we assume that the mode of transportation, used by the agent to perform each movement, is selected after the destination of the movement is chosen. However, the issue of choosing transportation mode is beyond the scope of this paper.

Stage 1:

For agents who have a daily routine, i.e. pupils, students, or working agents, the probability of moving on mornings of weekdays, is very high if their location is not their working place/school/university. Otherwise, each group of agents is characterized by a set of probabilities to move, depending on their location and time of day.

Stage 2:

After determining whether the agent is going to move (based on the probabilities mentioned above), the algorithm sets their destination. This is done based on the type of movement (routine/irregular) and the agents’ characteristics. If the agent has a regular routine and he is going to follow it, the location is predetermined and the agents goes there (i.e. school, university, working place). However, if they do not move towards their regular destination they can either stay home or move to a unique destination, this is determined based on different probabilities that represent the agents’ likelihood to move toward a specific land use, at each of the 6 time periods of a weekday. (see a detailed description of these probabilities in Appendix C in S1 File). These probabilities are different for each group, based on its characteristics. For example, the probability of a working family man to go to a public building in the morning rush hours is 2.5 times higher than the probability of a teenager to go there. On the other hand, the probability of a bachelor to go to an entertainment area at night is 25 times higher than the probability of a family man to go there at the same time. Outside the working/studying hours (e.g. afternoons or weekends) the destination is determined following two phases: first, based on the probabilities mentioned above, a land use is chosen. Then, for each cell *i* that is characterized by the chosen land use, we calculate the *IAC* (Integrated Attraction Coefficient) which is, in fact, an extended spatial gravitation parameter where the size is represented by the spatial size of the land use cluster. The *IAC* is therefore defined as follows:

$$IAC_{i}=d^{\delta_{i}}*g^{\gamma_{i}}*\theta_{i}*m_{i,rand}*f_{agent}$$ (1)

Where *d* represents the distance parameter (set here as 0.95), *g* represents the gravity parameter (set here as 1.02), *δ*_*i*_ is the distance from the agents’ current location to cell *i* (normalized), *γ*_*i*_ is the size of the cluster to which cell *i* belongs (in terms of spatial measurements), *θ*_*i*_ represents the specific attractiveness of cell *i*, *m*_*i*,*rand*_ is a random parameter, and *f*_*agent*_ represents the individual preferences of the examined agent (each agent has 40 locations in the city that he favors. These locations are randomly assigned to each agent. For the rest of the cells this value is set at unity).

To demonstrate how the IAC is calculated we can use the following example: if an agent is staying at a specific cell *j* at 8:30 PM and decides to move to a Public Open Area, the IAC value of another specific cell *i* (which is assigned with this land use), which has a specific attraction value of 0.93, is not one of this agent preferred locations, its normalized distance from cell *j* is 27.22, and is a part of a cluster with size of 29 cells, will be 0.42 in case of a temporal mood factor of 1.03 (*IAC*_16_ = 0.95^(27.22)^ * 1.02^29^ * 0.93 * 1.03 * 1 = 0.42).

All cells with the same land use are ranked, based on their IAC values and one cell is picked from the first 10 highest scores. By following this method, we assure that the destination is chosen for a unique agent in a unique time. Thus, an irregular movement is combined with the individual preferences of the agents.

## Results

We ran the model 20 times with 10,000 agents over a period of a month. While the agents were generated randomly in each run, the city configuration remained the same. We examine the results of the agents’ movement in several aspects: (1) distance distribution of the agents, (2) regularity of the movements and (3) attractiveness of specific locations. Based on the above, in this section we present results which represent typical examples for the qualitative behavior of the model.

### Distance distribution of all agents

**Fig 2:**
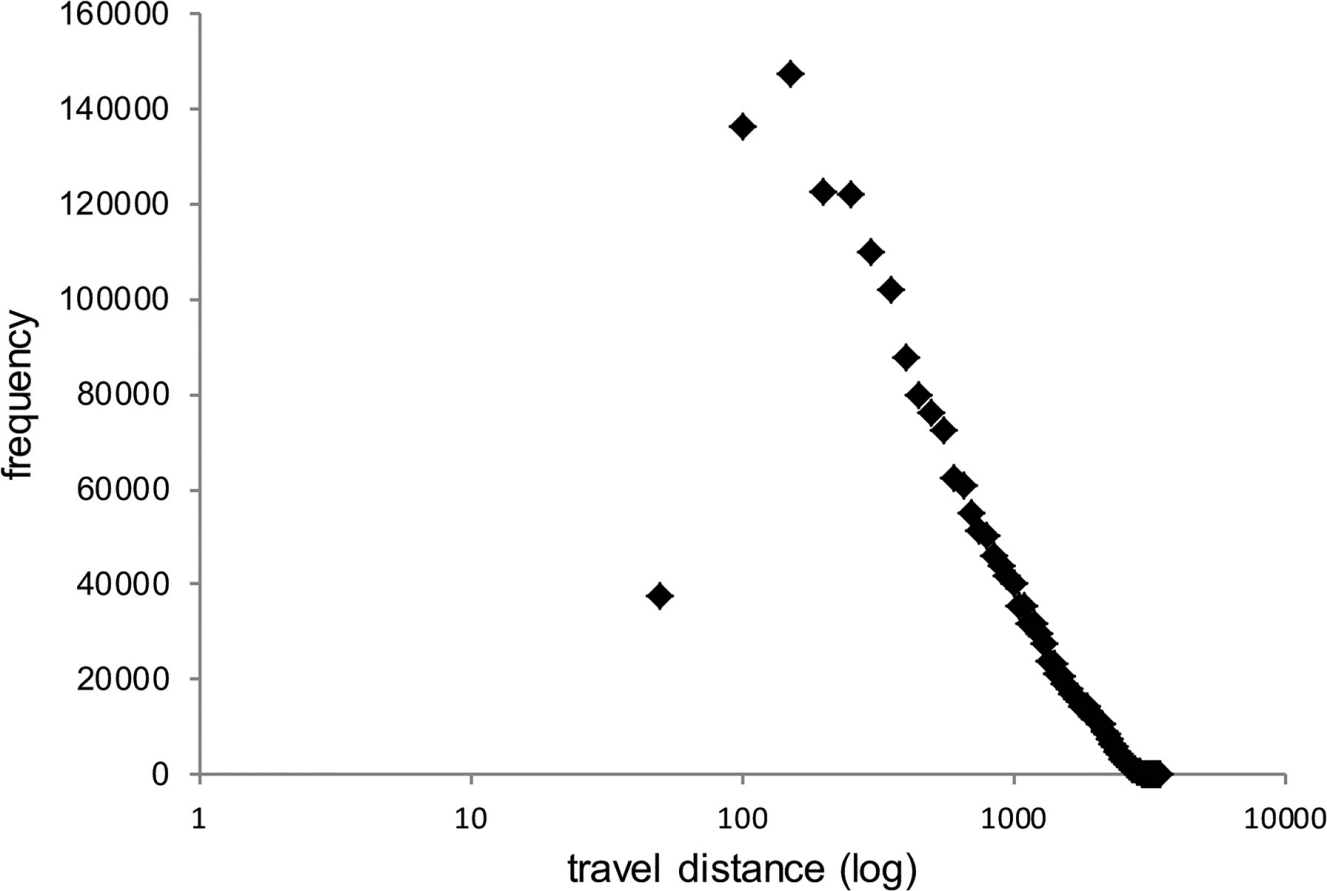
Frequency of distances for all the travels made during a month (model time) on semi-log scales.

**Fig 3:**
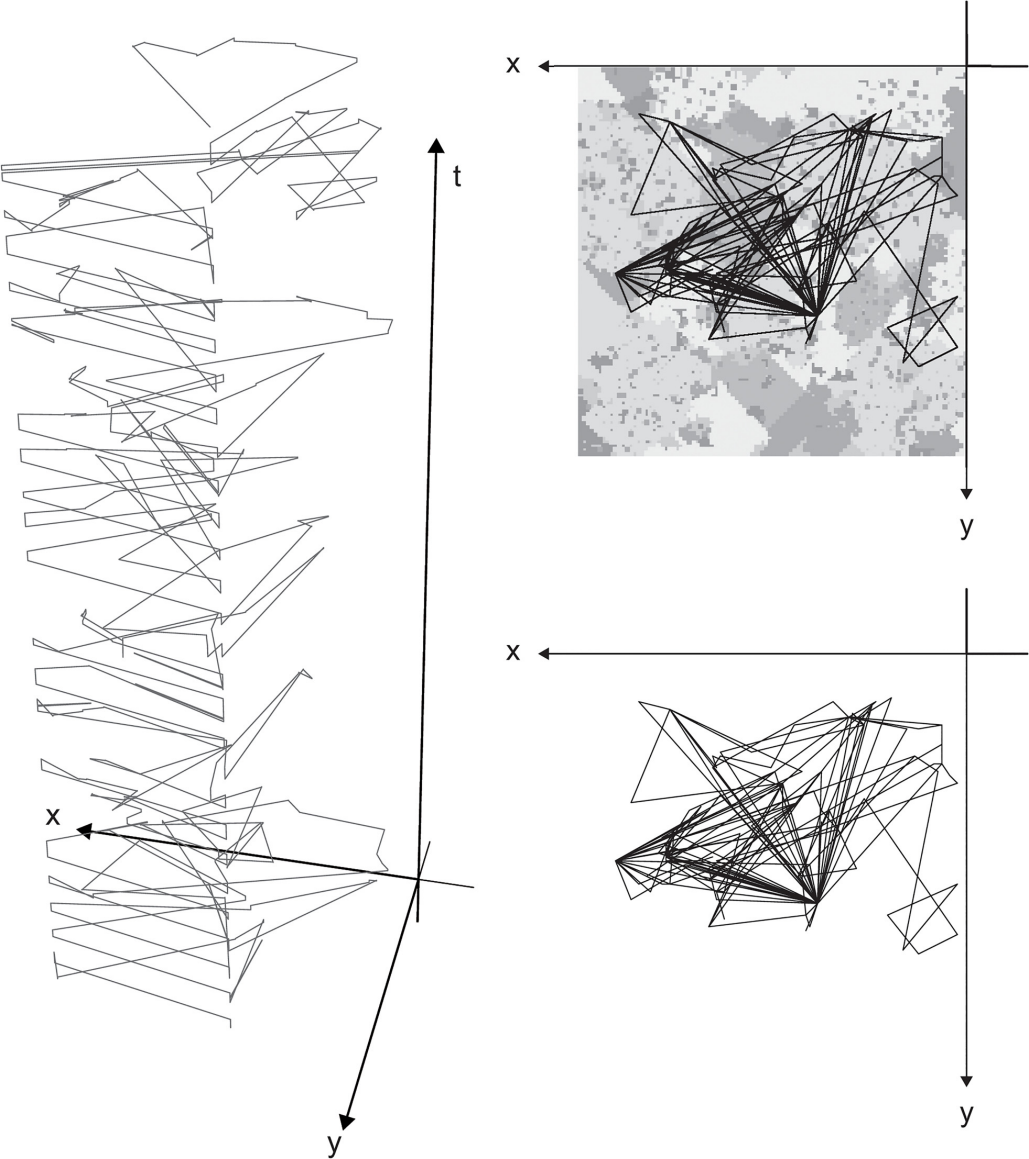
Spatio-temporal representation of an agent’s movement during one month. X and Y axes represent space while the Z axis represents time. The agent’s movements are represented by the lines. Note that there is on obvious regularity in the agent’s movement.

The results of the model suggest that the distribution of traveled distances (of all agents) obey a heavy-tailed distribution (see Fig 2). This means, that most of the movements the agents conducted were short-distance ones (less than 500 meters) while few travels were long-distance (above 2,500 meters). This result qualitatively aligns with real data analysis of human mobility patterns in urban environments as presented in [10, 13, 14]. An explanation for this finding (both in our model and in the empirical data) is that people travel between few main centers around which they make most of their movements. These centers are, for example, one’s home, work, school, etc. However, most of the movements people make are not between these centers but around them (see an example in Fig 3). To demonstrate this, we can take, for example, two typical people: a family man whose working place is located far from his home and a bachelor student. The family man leaves home in the morning and drives his children to school. Then, he goes to work where he spends most of his day. At lunchtime he goes to a nearby restaurant and when his working day is finished he might go to the supermarket that is located near his working place or near home. Then, he might pick his children up from a friend’s house or return home directly. In the evening he might go to a neighborhood café with his spouse. This scenario represents two long distance travels (children’s school – work, supermarket – children’s pick up) and 7 short distance travels (home – school, work – restaurant, restaurant – work, work – supermarket, children’s pick up – home, home – neighborhood café, neighborhood café – home). At the same time, the student leaves home in the morning. He stops to get coffee at the neighborhood café before continuing to his university where he goes to classes in 3 different buildings on the campus, and eats lunch at the student’s restaurant on campus. In the afternoon, he goes to work and spends a few hours there. Then, he goes to study at a friend’s house near his home and when they finish they go out together to the neighborhood bar. This scenario represents 3 long distance travels (café – university, university – work, work – friend’s house) and 6 short distance ones (home – café, 3 movements within the university campus, friend’s house – neighborhood bar, neighborhood bar – home). In these examples we find 5 long distance travels and 13 short distance ones. We can go on, of course, and demonstrate additional scenarios but we believe that these two examples represent the general behavior of people in the city.

### Regularity of the movements

In this section we present the results of the model concerning the regularity or irregularity of the agents’ movements. In other words, we examine how often each agent returns to specific locations. Empirical data found in [10] suggests that there are few locations people tend to return to on a regular basis, while there are many locations people visit seldom and irregularly. In our analysis, we examine about 10,000 agents over a one month period (model time). The number of different locations (cells) the agents visited varies between 31 and 124. The most visited cell hosted between 28 and 45 visits from the same agent over a month, where around 2,600 agents visited a unique location 45 times over that period. In the examined runs, all agents have more than one location, to which they returned 4 times or more, i.e. in addition to their home they had at least one more location they visited, probably on a weekly basis. 69% of all agents had at least two locations they returned to 20 times or more (which corresponds to the weekdays in a 4 week month), meaning they were students, pupils or employed.

**Fig 4:**
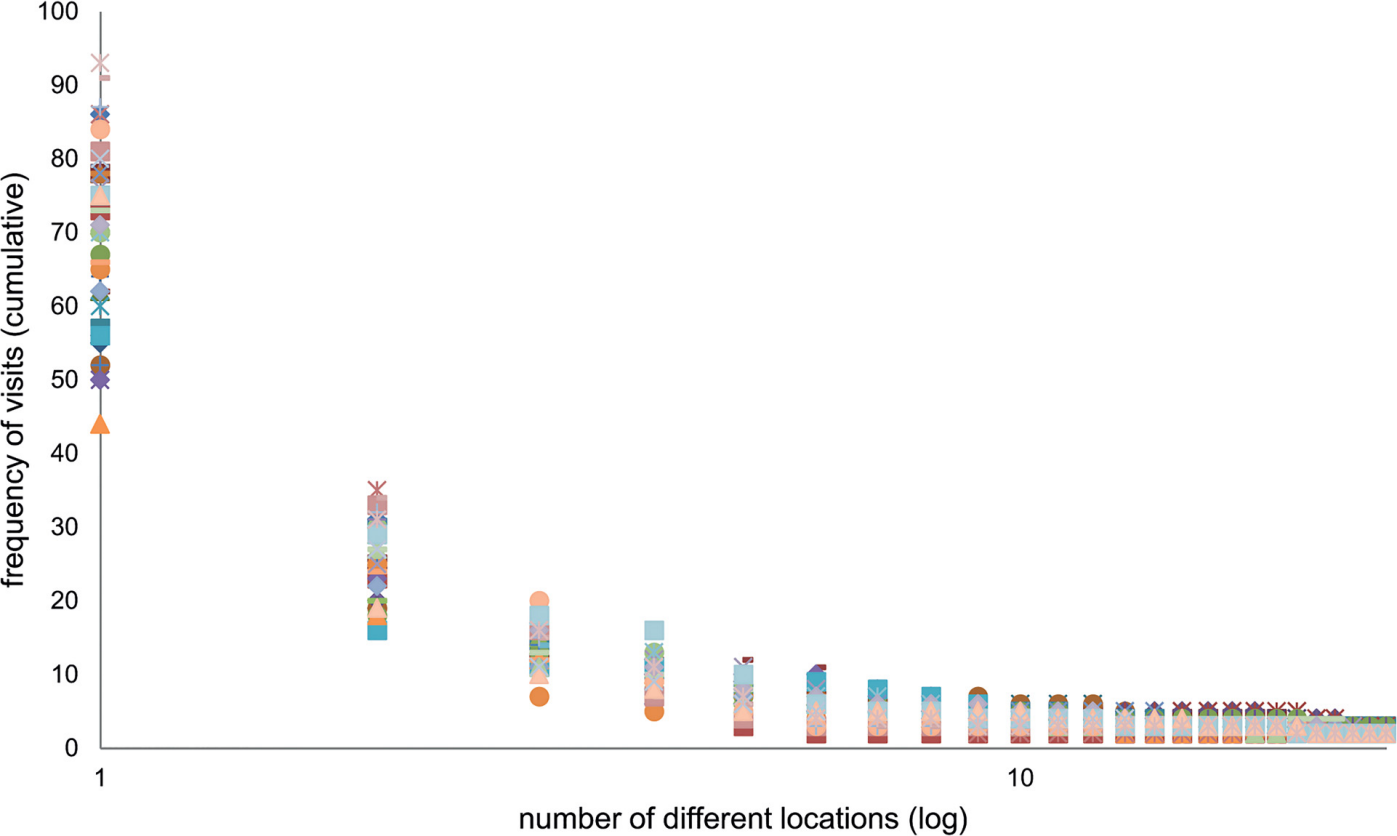
Cumulative frequency of visits to specific locations for 50 randomly selected agents on semi-log scales. Note that despite the qualitative similarity of the different agents’ behavior, there are quantitative differences among them.

Fig 4 presents the cumulative distribution of the returning of agents to the 25 most visited locations, for 50 randomly selected agents. As can be seen, this is a heavy-tailed distribution that corresponds to a power law. This suggests that here too, the results of our model correspond to real data [10]. These results show that each agent has few locations that he often visits and many locations that he rarely goes to. Additionally, the power law distribution suggests that the frequency of visits to unique locations is scale-invariant, meaning it is not dependent on the level of popularity of the cell (for a particular agent) and varies under the same rules for the most popular as well as the less attractive cells. This is, in fact, a self-organized attribute that emerged from the stochastic behavior of the agents. It implies that like in real urban systems, the collective behavior of the agents yields organized patterns that cannot be related to a particular parameter of the model.

### Attractiveness of specific locations

**Fig 5:**
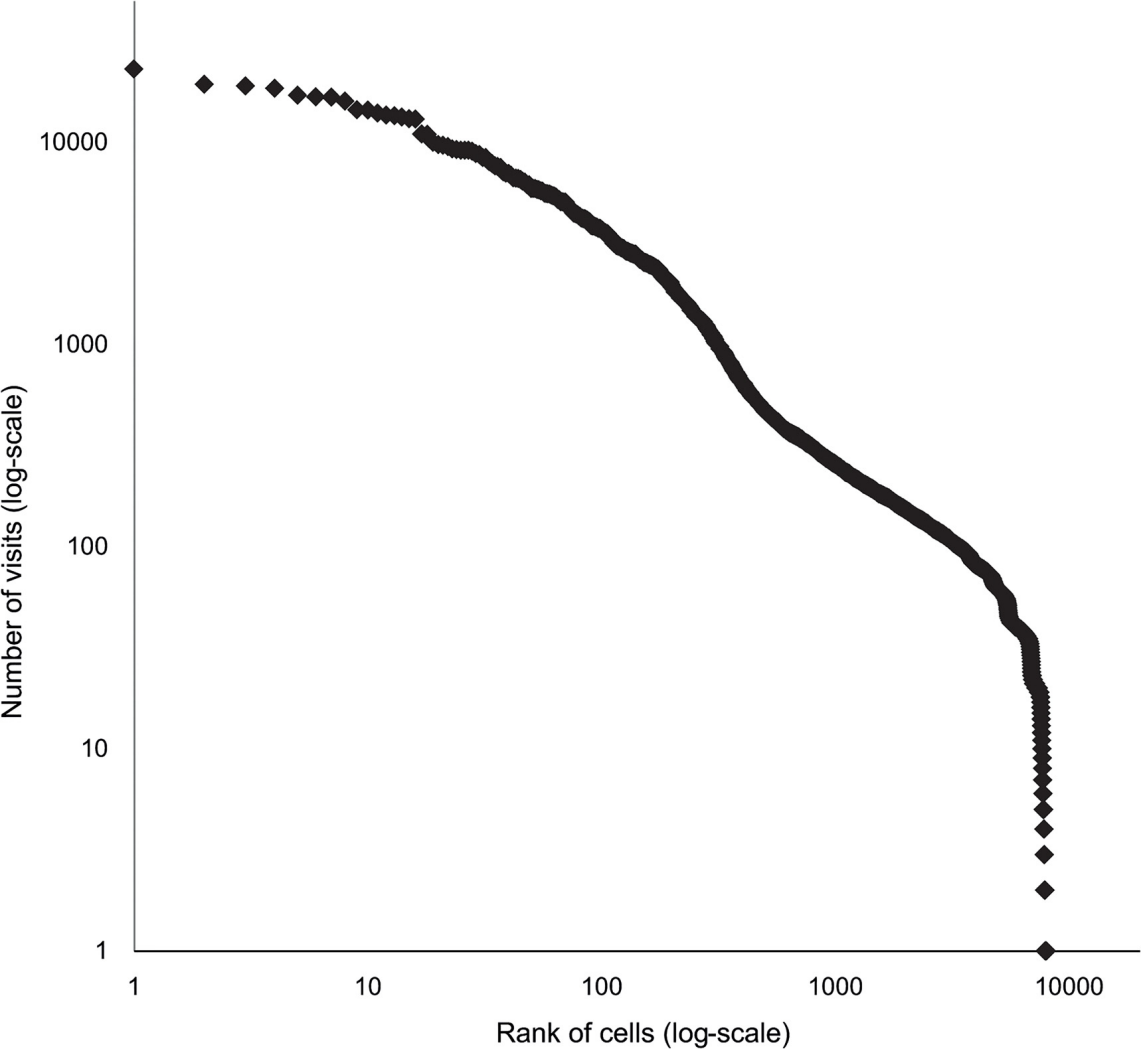
Rank size distribution of visit per cell over a month (model time).

Here, we focus on the attractiveness of specific locations to all agents in the model. Urban areas are not homogenous in terms of popularity. For all kind of land uses, some areas are more attractive than others. In our model, the attractiveness of a cell is not predetermined as the IAC of each cell is different for each agent as it is depended on the agent’s characteristics, location, the time of day, and a random parameter. Nevertheless, the results of the model show that some locations are far more attractive than others. Fig 5 presents the rank size distribution of visits per cell. This graph shows the frequency of visits to each cell as a dependent of the cell’s rank (the most popular cell is ranked 1, the second most popular cell is ranked 2 and so on). It can be seen, that while the most popular cell hosted about 23,000 visits during the measured period (one month – model time), most cells hosted a considerably smaller number of visits during the same period. The average number of visits per cell was 207 and around 2,000 cells were never visited. Looking at the distribution of the cells’ popularity we find a heavy tail distribution. While 96.9% of the cells were visited between 0–1,000 times, 2.9% were visited between 1,000–10,000 times and only 18 cells (which represent 0.2% of the cells) were visited more than 10,000 times. Thus, we can divide the cells into 3 groups, based on their popularity: group A: the most popular cells (18 cells, each was visited more than 10,000 times per month), groups B: popular cells (293 cells, each was visited between 1,000–10,000 times per month), and group C: the majority of cells (9,687 cells, each was visited less than 1,000 times per month).

**Fig 6:**
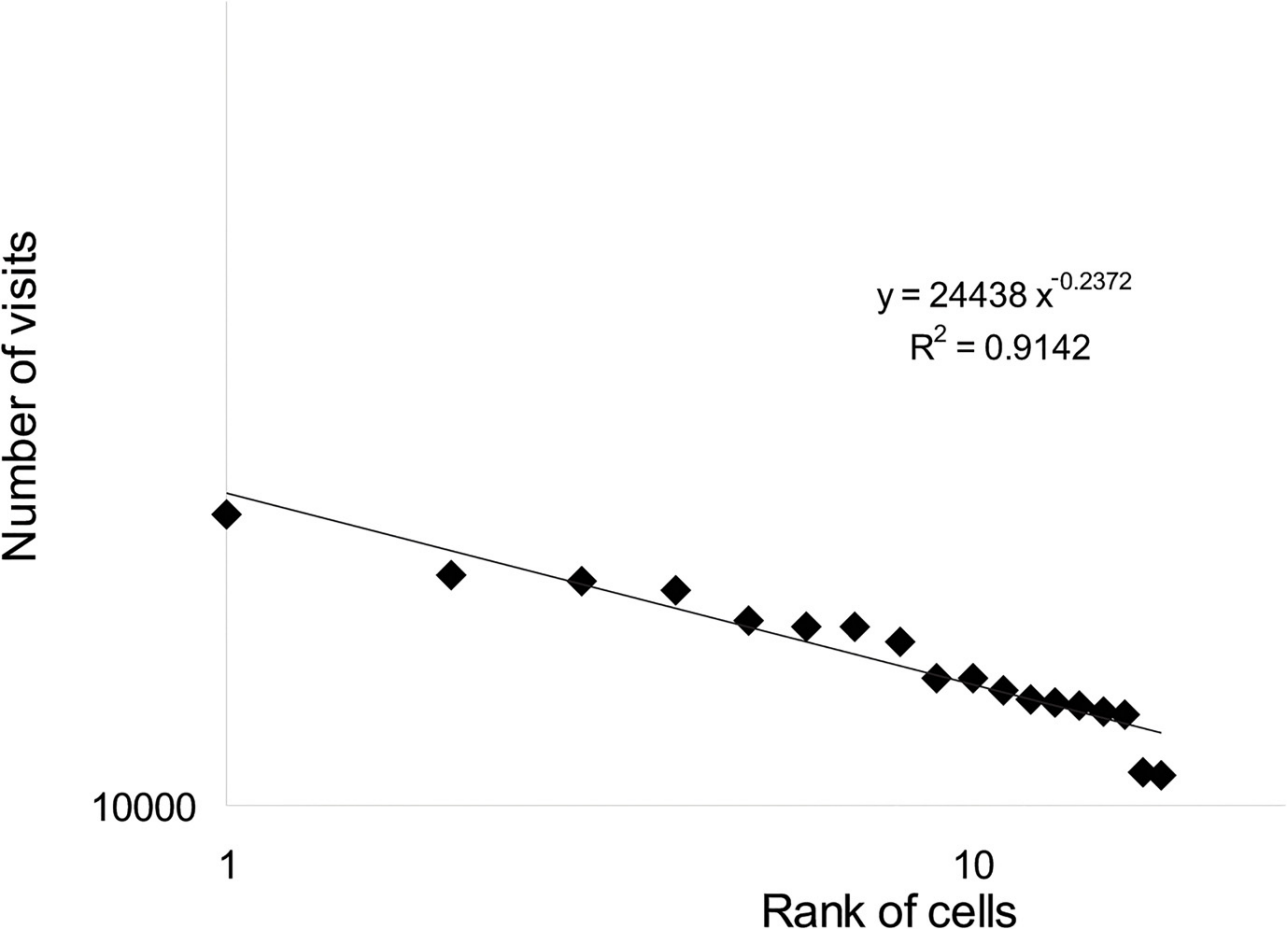
Rank size distribution of group A.

We wanted to examine the distribution of visits per cell for the above 3 groups. However, as group A consist of only 18 cells, we present the rank size distribution instead. Both distributions can actually be replaced with each other (see [36] for a detailed explanation). When looking at group A we can see that the ranks size distribution of visits per cell follows a heavy tail distribution that fits a power law (Fig 6). An inspection of the cells that belong to group A, revealed that the land uses of these cells are any of the possible land-uses (see Appendix D in S1 File for a detailed description of land use combinations for each group, in different runs of the model). The sizes of the clusters to which these cells are associated also didn’t reveal any significant quality as they range from small cluster (50 cells or less) to large ones (950 cells). The common characteristic of these cells, however, is their spatial location; all of them were located on (or in proximity to) boundaries between different land uses. These results hold for different runs of the model and for different spatial configurations.

**Fig 7:**
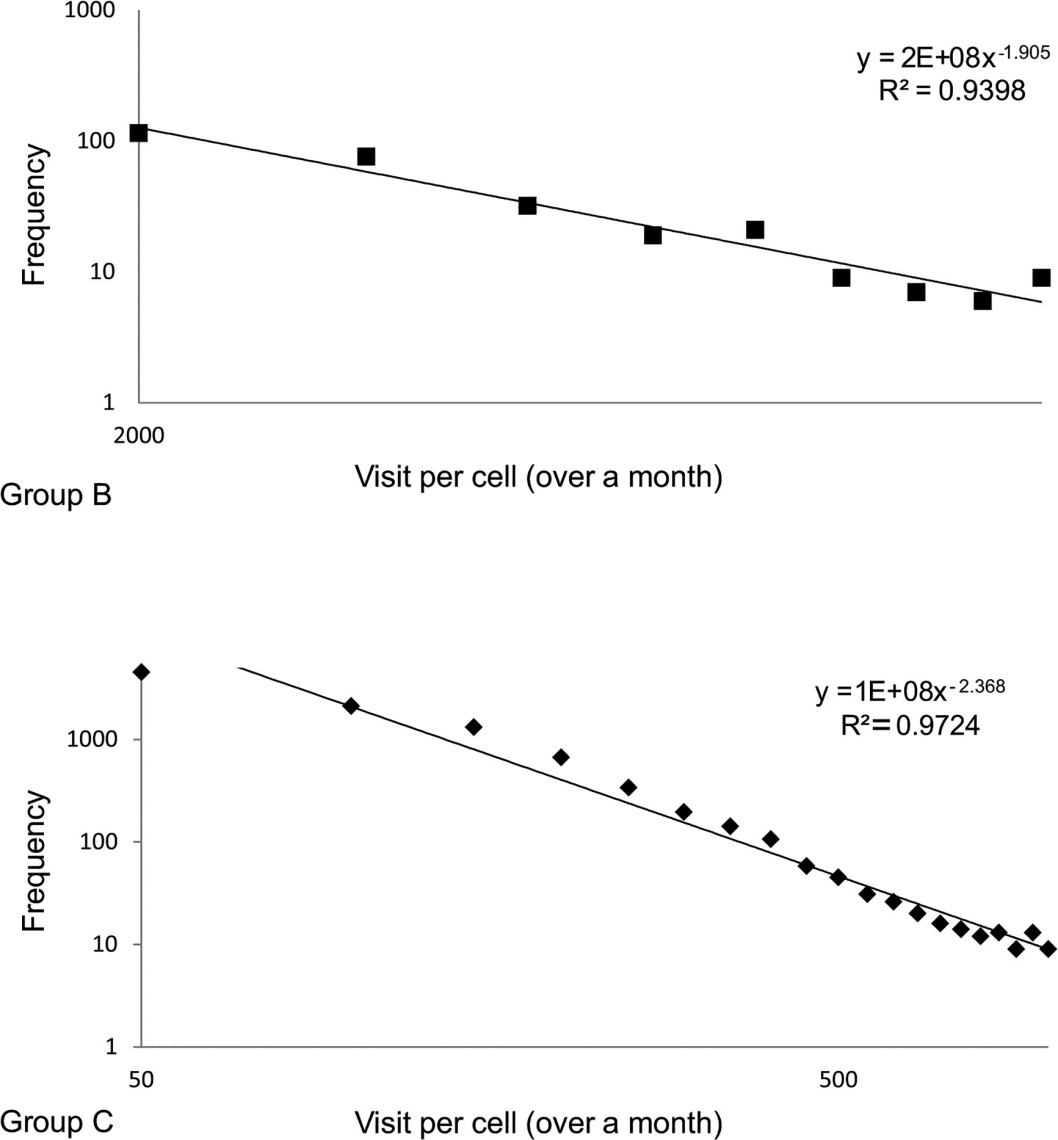
Distribution of visits per cell for group B and group C.

The distributions of visits per cell for groups B and C can be seen in Fig 7. Both distributions also follow a power law but each with different parameters. The scale-free characteristics of all three distributions can indicate that even though the system as a whole corresponds to three different types of behavior, each of these behaviors follow a clear trend that is continuous for all levels of popularity the cells exhibits.

## Summary

In this work we presented a spatio-temporal agent-based model for human mobility patterns. This is a bottom-up, stochastic model that is based on the behavior of 10,000 agents, where their movement imitates real-life behavior, and depends on several attributes, among them are the attraction to a specific location and the distance from it (i.e. spatial gravitation). The results of our model correspond to empirical data on human mobility patterns in the intra-urban scale. This is achieved by combining some basic characteristics of human agents such as their employment status, marital status, personal preferences, etc., and in particular, the consideration of regular and irregular trajectories, along with the gravitation model. Note, that the regularity of the agent’s movement is not a pre-determined (top-down) one, but results from the probabilities that affect the agent’s decision to move, and where to move to. Our results fit the movement of the agents themselves in terms of individuals and of the entire population, as well as to spatial dynamics of the urban environment, i.e. the emergence of popular locations in the city due to bottom up human behaviors that are not governed by any top-down forces. This suggests that by combining the gravitation model with additional considerations, it can be applied to the city’s scale as well as to larger scales (as shown by [21]).

The presented model is innovative and significant as it is based on the assumption that HMP are space-dependent and follow high regularity. Although these assumptions are known in the field of urban geography since the 1960s, they have not been implemented in urban simulation models of this type. To the best of our knowledge this is the first time simulating human mobility patterns at the intra-urban scale was carried out successfully. Thus, it holds a potential to be used as the basis of many urban simulations which rely on human mobility. This model allows isolating specific parameters of the complex reality, thus revealing the effects of their mutual effect and interdependencies. By modeling situations that are being observed in real cities, we can expand our understandings on HMP, and therefore improve urban planning, transportation planning, epidemiology models, mobile wireless network planning and more.

## Supporting Information

S1 File Appendix. Appendix A in S1 File. The algorithm that determines whether the agent is going to move or stay put. Appendix B in S1 File. The algorithm that sets the agent’s destination (once it has been determined that the agent is indeed moving). Appendix C in S1 File. The probabilities that represent the agents’ likelihood to move toward a specific land use, at each of the 6 time periods of a weekday. Appendix D in S1 File. A description of land use combinations for each group, in different runs of the model
(ZIP)
