# Supplementary figures and images for: A Simulation Model for Intra-Urban Movements
Source: PLoS One. 2015 Jul 10;10(7):e0132576. doi: 10.1371/journal.pone.0132576 (PMC4498912; doi:10.1371/journal.pone.0132576)

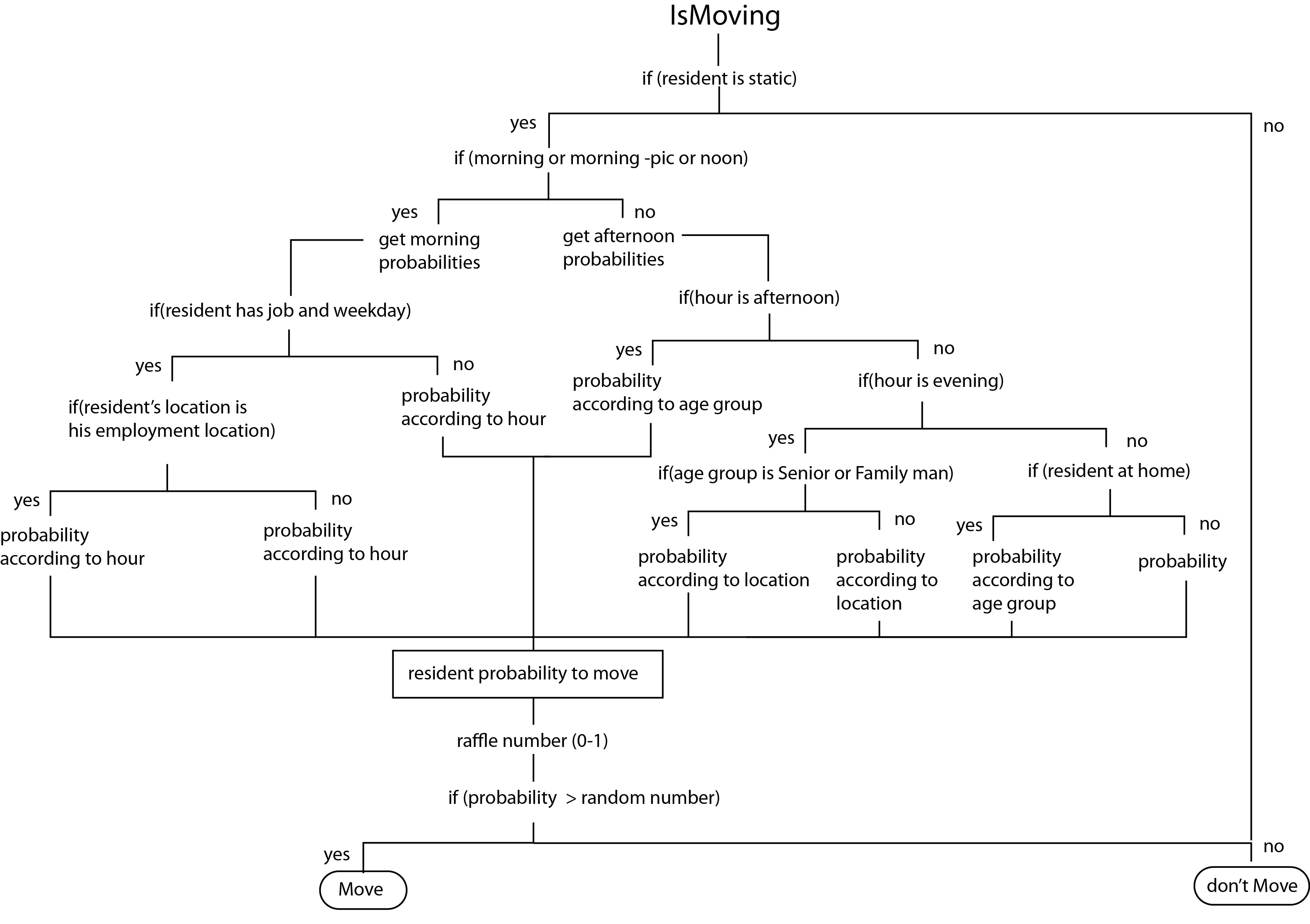

Supplement: S1 File — Appendix A in S1 File. The algorithm that determines whether the agent is going to move or stay put. Appendix B in S1 File. The algorithm that sets the agent’s destination (once it has been determined that the agent is indeed moving). Appendix C in S1 File. The probabilities that represent the agents’ likelihood to move toward a specific land use, at each of the 6 time periods of a weekday. Appendix D in S1 File. A description of land use combinations for each group, in different runs of the model (ZIP) [file pone.0132576.s001.zip › S1/AppendixA.jpg]

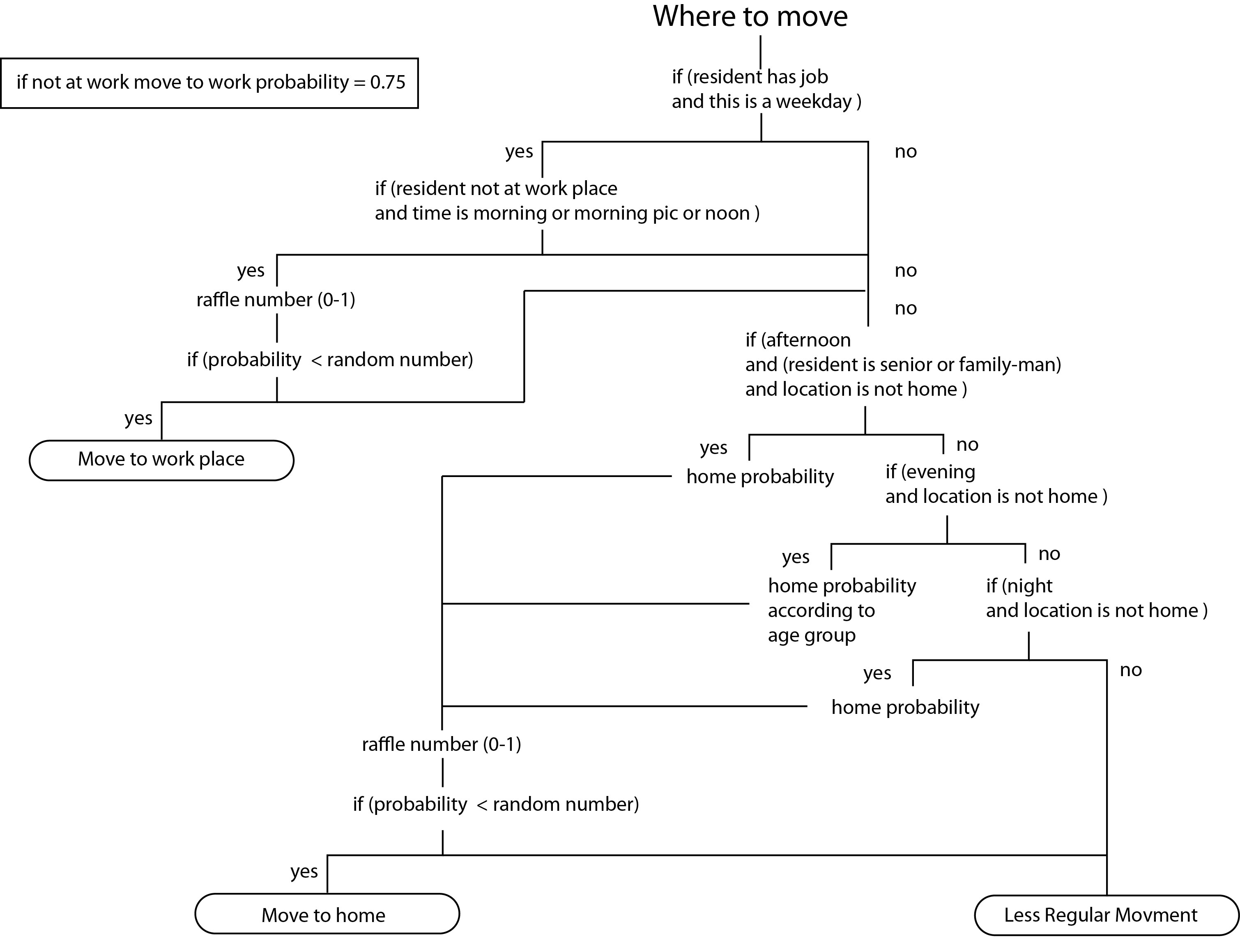

Supplement: S1 File — Appendix A in S1 File. The algorithm that determines whether the agent is going to move or stay put. Appendix B in S1 File. The algorithm that sets the agent’s destination (once it has been determined that the agent is indeed moving). Appendix C in S1 File. The probabilities that represent the agents’ likelihood to move toward a specific land use, at each of the 6 time periods of a weekday. Appendix D in S1 File. A description of land use combinations for each group, in different runs of the model (ZIP) [file pone.0132576.s001.zip › S1/AppendixB.jpg]

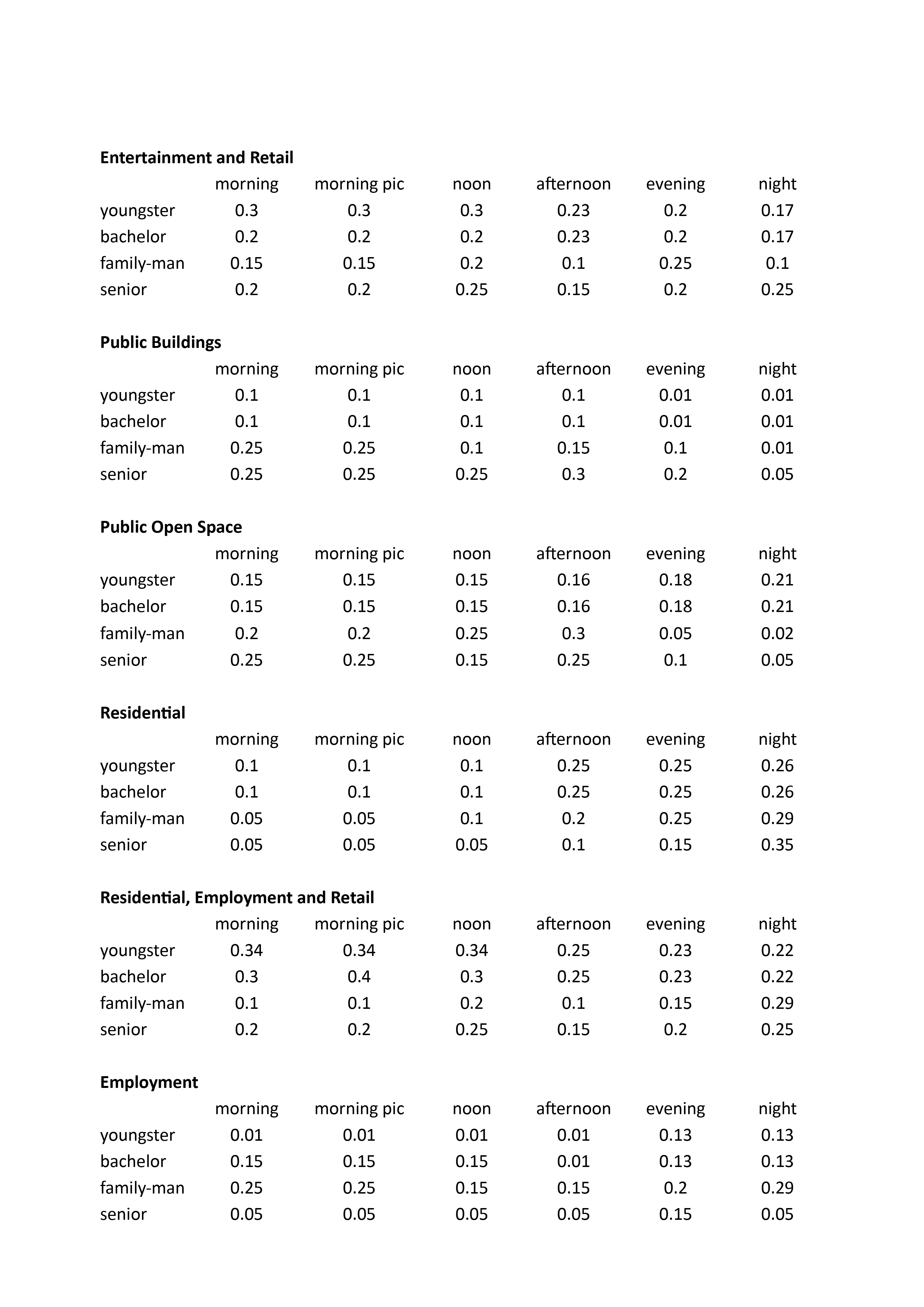

Supplement: S1 File — Appendix A in S1 File. The algorithm that determines whether the agent is going to move or stay put. Appendix B in S1 File. The algorithm that sets the agent’s destination (once it has been determined that the agent is indeed moving). Appendix C in S1 File. The probabilities that represent the agents’ likelihood to move toward a specific land use, at each of the 6 time periods of a weekday. Appendix D in S1 File. A description of land use combinations for each group, in different runs of the model (ZIP) [file pone.0132576.s001.zip › S1/AppendixC.jpg]

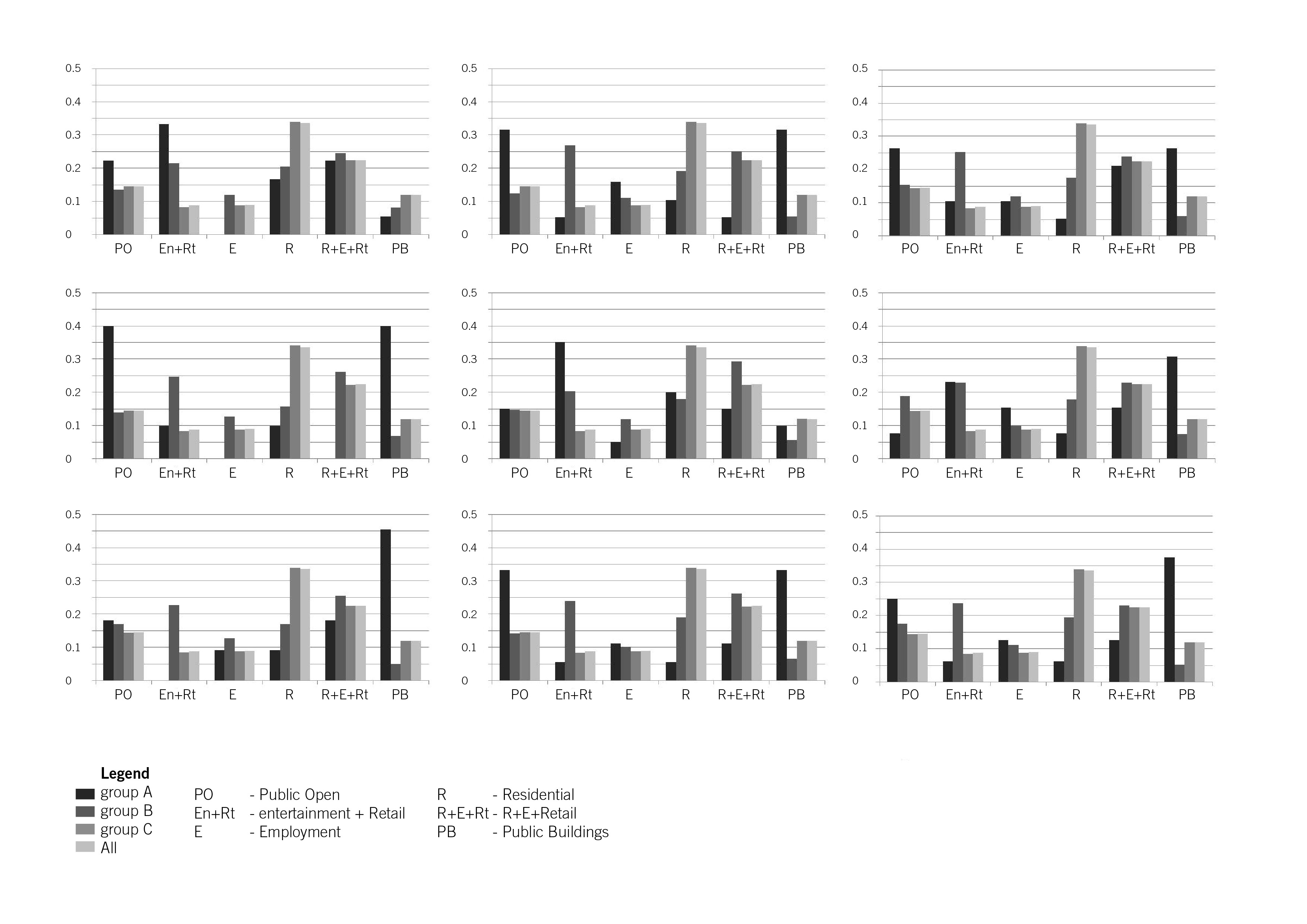

Supplement: S1 File — Appendix A in S1 File. The algorithm that determines whether the agent is going to move or stay put. Appendix B in S1 File. The algorithm that sets the agent’s destination (once it has been determined that the agent is indeed moving). Appendix C in S1 File. The probabilities that represent the agents’ likelihood to move toward a specific land use, at each of the 6 time periods of a weekday. Appendix D in S1 File. A description of land use combinations for each group, in different runs of the model (ZIP) [file pone.0132576.s001.zip › S1/AppendixD.jpg]
